# Supplementary material for: The genome sequence of the Heath Bumblebee, Bombus jonellus (Kirby, 1802)
Source: Wellcome Open Res. 2025 May 23;10:269. [Version 1] doi: 10.12688/wellcomeopenres.24256.1 (PMC12166357; doi:10.12688/wellcomeopenres.24256.1)
Supplement: Peer review history

# Reviewer report: Reviewer response for version 1

**DOI:** 10.21956/wellcomeopenres.26753.r124962

**Published:** 2025-06-13

**Authors:** Erich D Jarvis

**Affiliations:** 1 Rockefeller University, Millbrook, USA

This is a good genomic data note. It is well written, explained well, with good figures. The genome assembly is good. Not sure why the authors did not use the Hi-C phasing mode to get two relatively complete haplotypes. In future assemblies, it would be good to do so. No changes though are necessary for the current Data Note

Are sufficient details of methods and materials provided to allow replication by others?

Yes

Is the rationale for creating the dataset(s) clearly described?

Yes

Are the datasets clearly presented in a useable and accessible format?

Yes

Are the protocols appropriate and is the work technically sound?

Yes

Reviewer Expertise:

Genomics and Neuroscience

I confirm that I have read this submission and believe that I have an appropriate level of expertise to confirm that it is of an acceptable scientific standard.

# Reviewer report: Reviewer response for version 1

**DOI:** 10.21956/wellcomeopenres.26753.r124303

**Published:** 2025-05-30

**Authors:** Jason Charamis

**Affiliations:** 1 Foundation for Research and Technology - Hellas, Irákleion, Greece

This data note presents a high-quality genome assembly of the Heath Bumblebee (Bombus jonellus), contributing valuable genomic resources to the Darwin Tree of Life project. The technical work is solid and the manuscript is generally well-written.

 The assembly achieves good quality metrics with 357.90 Mb total length and 78.06% scaffolded into 18 chromosomes, while it also has a high BUSCO completeness score (97.6%) with low duplication rate.

 I have only two comments to make:

 1. The introduction would benefit from brief discussion of why the B. jonellus genome is scientifically important beyond its contribution to the Darwin Tree of Life project.

 2. The manuscript does not discuss broader implications of this genomic resource in ecological studies.

Are sufficient details of methods and materials provided to allow replication by others?

Yes

Is the rationale for creating the dataset(s) clearly described?

Yes

Are the datasets clearly presented in a useable and accessible format?

Yes

Are the protocols appropriate and is the work technically sound?

Yes

Reviewer Expertise:

Arthropod Comparative Genomics

I confirm that I have read this submission and believe that I have an appropriate level of expertise to confirm that it is of an acceptable scientific standard.
